# A Probiotic Adjuvant *Lactobacillus rhamnosus* Enhances Specific Immune Responses after Ocular Mucosal Immunization with Chlamydial Polymorphic Membrane Protein C

**DOI:** 10.1371/journal.pone.0157875

**Published:** 2016-09-16

**Authors:** Aleksandra Inic-Kanada, Marijana Stojanovic, Emilija Marinkovic, Elisabeth Becker, Elisabeth Stein, Ivana Lukic, Radmila Djokic, Nadine Schuerer, Johannes H. Hegemann, Talin Barisani-Asenbauer

**Affiliations:** 1 OCUVAC – Center of Ocular Inflammation and Infection, Laura Bassi Centres of Expertise, Center for Pathophysiology, Infectiology and Immunology, Medical University of Vienna, Vienna, Austria; 2 Department of Research and Development, Institute of Virology, Vaccines and Sera – TORLAK, Belgrade, Serbia; 3 Institut für Funktionelle Genomforschung der Mikroorganismen, Heinrich-Heine-Universität Düsseldorf, Universitätsstr. 1, Gebäude 25.02.U1, 40225, Düsseldorf, Germany; University of the Pacific, UNITED STATES

## Abstract

Recent advances in the development of chlamydia vaccines, using live-attenuated or ultraviolet light-inactivated chlamydia, are paving the way for new possibilities to oppose the societal challenges posed by chlamydia-related diseases, such as blinding trachoma. An effective subunit vaccine would mitigate the risks associated with the use of a whole-cell vaccine. Our rationale for the design of an efficient subunit vaccine against *Chlamydia trachomatis* (Ct) is based on the membrane proteins involved in the initial Ct-host cell contact and on the route of immunization that mimics the natural infection process (i.e., via the ocular mucosa). The first aim of our study was to characterize the specific conjunctival and vaginal immune responses following eye drop immunization in BALB/c mice, using the N-terminal portion of the Ct serovar E polymorphic membrane protein C (N-PmpC) as the subunit vaccine antigen. Second, we aimed to examine the adjuvant properties of the probiotic *Lactobacillus rhamnosus* (LB) when formulated with N-PmpC. N-PmpC applied alone stimulated the production of N-PmpC- and Ct serovar B-specific antibodies in serum, tears and vaginal washes, whereas the combination with LB significantly enhanced these responses. The N-PmpC/LB combination initiated a T cell response characterized by an elevated percentage of CD25+ T cells and CD8+ effector T cells, enhanced CD4+ T-helper 1 skewing, and increased regulatory T cell responses. Together, these results show that eye drop vaccination with combined use of N-PmpC and a live probiotic LB stimulates specific cellular and humoral immune responses, not only locally in the conjunctiva but also in the vaginal mucosa, which could be a promising approach in Ct vaccine development.

## Introduction

Eye drop vaccination via the ocular mucosa constitutes an attractive immunization approach, particularly for the prevention/treatment of ocular infections and their blinding sequelae [1, 2]. Furthermore, ocular topical immunization is safer than nasal immunization because there is no risk of antigen redirection towards the nervous system [1] and is needle-free, which is more comfortable and safer than parenteral vaccination [3].

*Chlamydia trachomatis* (Ct) is a human pathogen causing chronic conjunctivitis and is also the most common cause of sexually transmitted disease. Ct infections can be asymptomatic and, if left untreated, can result in blinding trachoma (the leading cause of preventable blindness worldwide) and pelvic inflammatory disease, which can lead to infertility and ectopic pregnancy [4–6]. Regardless of the infection site, there is a consensus that a vaccine is needed [7–11]. Even a partially effective vaccine would contribute in reducing the global disease burden caused by Ct [12].

Polymorphic membrane proteins (Pmps) are essential in early *Chlamydia*-host cell interactions [13–15] and are recognized as potential vaccine antigens to inhibit both contact and infection [15]. Furthermore, a relatively high portion of the Ct genome encodes for Pmp superfamily proteins [16], suggesting their importance in the *Chlamydia* life cycle [17]. Epitope mapping of the PmpC region encompassing the amino acid residues 605–840 demonstrated a broad B cell recognition range. In addition, the full-length protein was shown to react with the serum from Ct-infected minipigs [18]. We have previously shown that N-PmpC can trigger heterologous immunity as well as the positive influence of particulate Gram-negative bacterial adjuvants on the development of antigen-specific immune responses after topical ocular immunization [19].

Available data suggest a hyporesponsiveness of conjunctiva-associated immune cells to lipopolysaccharide stimulation [20] and an important role for TLR2 agonists in the abrogation of the immunosuppressive mechanisms naturally occurring within the conjunctiva [21]. These findings led to the hypothesis that Gram-positive bacteria, e.g., probiotic *Lactobacillus rhamnosus* (LB), which are also corpuscular in nature, may be employed as an efficient adjuvant when immunizing via the conjunctiva. Furthermore, *Lactobacillus* spp. (*Lactobacillus acidophilus*) was previously used in ocular surface applications without signs of toxicity or safety risks [22].

The aim of our study was to characterize the local and systemic immune responses produced by the chlamydia-specific subunit antigen N-PmpC by analysing serum and mucosal washes from *Chlamydia-*relevant surfaces (i.e., conjunctiva and vagina). In addition, we sought to assess the modulatory effect of the probiotic bacterial adjuvant LB on the post-immunization immune response.

## Materials and Methods

### Ethics statement

All experiments were approved by the "Ethics Committee for the Welfare of Experimental Animals" and by the committee section at the Institute of Virology, Vaccines and Sera–TORLAK. All experiments conformed to the Serbian laws and European regulations on animal welfare (Approval No. 011-00-00510/2011-05). Every effort was made to minimize animal suffering. Mice that were immunized were anesthetized by intraperitoneal (i.p.) administration of a mixture of xylazine (Sigma-Aldrich, Kansas, KS, USA) and ketamine (Richter Pharma AG, Wels, Austria). The method for mice euthanasia was cervical dislocation. We did not observe any unexpected deaths of animals during this study.

### Animals

Ten-week-old BALB/c female mice (six mice per group) were housed at the Animal Facility of the Institute of Virology, Vaccines and Sera (TORLAK) and kept at a temperature of 21°C under a 12:12 h light: dark cycle with *ad libitum* access to water and food.

### Antigens and adjuvants

A recombinant N-PmpC protein fragment (1–565 amino acids) from Ct serovar E produced in *E*. *coli* [15, 23] was used as the antigen.

LB (characterized by 16S rRNA sequencing, NCIMB Ltd, Aberdeen, UK, ref.no. NCSQ 18723; TORLAK) was used as the adjuvant. During preliminary validation experiments, LB was administered via the conjunctiva (5 μl/eye) at a concentration of 1 x 10^8^ CFU/ml, resulting in an LB immunization dose of 1 x 10^6^ CFU/dose. Additional preliminary results with different LB doses (1x10^9^ CFU/ml, 1x10^8^ CFU/ml, 1x10^7^ CFU/ml, 1x10^6^ CFU/ml) on days 0, 14 and 28 applied topically revealed no visible signs of inflammation or infection at the ocular surface, where signs of ocular irritation were monitored in all mice on a daily basis (during the course of immunization). Two “blinded” ophthalmologists, using magnifying loupes, assessed conjunctival hyperemia, edema and corneal clarity.

### Immunization schedules

Ten-week-old BALB/c female mice (n = 6 mice per group) were immunized with N-PmpC on days 0, 14 and 28, and the resulting immune responses were evaluated two weeks after the last immunization. The mice were immunized via the conjunctiva (conj//) with N-PmpC alone in PBS (conj//N-PmpC) or N-PmpC combined with LB at 1 x 10^8^ CFU/ml in PBS (conj//N-PmpC/LB). The concentration of N-PmpC in all vaccines was 1.5 mg/ml. Each mouse was immunized with 15 μg N-PmpC, with or without a 10^6^ CFU dose of LB, in a total volume of 10 μl (5 μl/eye, both eyes were treated). A group of age-matched non-immunized mice was used as the normal control group (nc). Our experimental procedure did not cause any visual impairment (including blindness) in animals, which was examined by an ophthalmologist during the whole immunization period. Furthermore, we did not observe any changes in behavioural pattern between treated animals and their respective controls.

### Sample collection

Blood serum samples were collected from the mouse tail vein (6 individual sera from 6 animals per group) two weeks after completion of the indicated immunization protocol. Wash samples were obtained two weeks after completion of the indicated immunization protocol by lavage with 15 μl of PBS for each eye for tear-wash samples, and with 150 μl for vaginal-wash samples.

### Detection of PmpC-specific and CtB-specific immunoglobulins

Quantification of N-PmpC- and Ct serovar B (CtB)-specific antibodies in sera, tear and vaginal washes was performed as previously described [19], slightly modified. Briefly, ELISA plates (MaxiSorp; Nunc, Roskilde, Denmark) were coated (50 μl/well) with N-PmpC (10 μg/ml N-PmpC in PBS) or CtB (1 x 10^6^ IFU/ml in PBS) by overnight adsorption at 4°C. Appropriately diluted non-pooled sera (1:100), tear or vaginal washes (1:10) were used as samples. Specific antibody levels in wash samples were expressed as a relative amount, calculated as the concentration of specific antibody in a particular sample divided by the lowest concentration of the same antibody in the corresponding nc sample.

### Lymphocyte phenotyping by FACS analysis

Submandibular lymph nodes (SMLN) from mice immunized via the ocular conjunctivae or control mice were aseptically isolated as previously described [19].

SMLN cells (1 x 10^6^ cells/sample) were immunostained using fluorochrome-conjugated antibodies specific for murine CD3 (FITC-conjugated, eBioscience, San Diego, CA), CD4 (PE-conjugated, Biolegend, San Diego, CA), CD8 (PerCP-conjugated, Biolegend), CD19 (PECy5-conjugated, eBioscience), CD25 (PE-conjugated, Biolegend or PECy5-conjugated, eBioscience) and Foxp3 (Alexa488-conjugated, Biolegend). Before staining, the cells were washed in a cold 2% BSA/0.1% NaN_3_/PBS solution (2 x centrifugation at 300 g, 5 min, 4°C). Fluorochrome-conjugated antibodies were added to the resuspended cell pellets and incubated in the dark for 30 min at 4°C. Discrete aliquots of each analysed cell suspension were incubated with the corresponding isotype control antibodies and used as the unstained reference for setting the FACS analysis staining thresholds. Unbound antibodies were removed by washing in cold 2% BSA/0.1% NaN_3_/PBS solution (3 x centrifugation at 300 g, 5 min, 4°C).

For the intracellular Foxp3 staining, the cells were first stained with anti-mouse CD4-PE and anti-mouse CD25-PECy5 and then fixed and permeabilized using the BD Cytofix/Cytoperm Buffer and BD CytopermPermeabilization Buffer Plus (BD Biosciences, San Jose, CA, USA), respectively, according to the manufacturer’s instructions. Washes between all steps were performed using the Perm/Wash Buffer (BD Biosciences; 3 x centrifugation at 300 g, 5 min, 4°C). An anti-mouse Foxp3-Alexa488 monoclonal antibody was added to the resuspended cell pellets and incubated in the dark for 30 min at 4°C. Unbound antibodies were removed by centrifugation (washing).

Stained cells were analysed using the BD FACScan^™^ flow cytometer (BD Biosciences). BD CellQuest^™^ software was used for analysis.

### Proliferation assay

Evaluation of proliferative response of SMLN cells upon stimulation with N-PmpC (10 μg/ml) and CtB (1 x 10^6^ IFU/ml) was performed as previously described [19]. Briefly, SMLN cells were plated into 96-well plates (100 μl/well, 2 × 10^6^ cell/ml in 10% FCS/50 μM β-mercaptoethanol/RPMI 1640) and incubated for 48 h (5% CO2, 37°C) without additional stimulation or in the presence of the stimulators. Cell Counting Kit-8 reagent (10 μl/well, Sigma Aldrich) was added upon 48h long incubation, and the cells were incubated for additional 4h. Reactions were stopped by the addition of 1% (w/v) sodium dodecyl sulfate (10 μl/ well), and absorbance values were measured at 450/650 nm (A_450/650_) using a spectrophotometer (Ascent 6–384 [Suomi], MTX Lab Systems Inc., Vienna, VA, USA).

The number of viable cells per well was calculated using a standard curve A_450/650_ = *f*(number of cells). Discrete pool of non-stimulated cells was used as standard after counting in the presence of trypan blue (Countess Automated Cell Counter, Invitrogen). Standard suspension was plated in serial dilutions prior to centrifugation and further treated identically as the experimental wells.

A proliferation index (PI) for each specifically stimulated cell suspension was calculated per individual animal. The PI index was defined as the ratio of number of viable cells per well present in stimulated (S) to number of viable cells per well present in corresponding non-stimulated (So) cultures, such that PI = Ss/So.

### SMLN cell cytokine profiling

Production of IFN-γ, IL-4, IL-17A and IL-10 was analysed by measuring their concentrations in the supernatants of non-stimulated, N-PmpC- (10 μg/ml) and CtB-stimulated (1 x 10^6^ IFU/ml) SMLN cultured cells (2 x 10^6^ cell/ml in 10% FCS/50 μM β-mercaptoethanol/RPMI 1640; 37°C, 5% CO_2_, 48 h) using sandwich ELISA with commercially available monoclonal antibodies (eBioscience) [19].

### Statistical analyses

The statistical significance of the observed differences was evaluated using Kruskal-Wallis test followed by Dunn's multiple comparisons test to compare between groups. All statistical analyses were performed with the GraphPad 6.0 software. A probability (*P*) value of 0.05 was set as the significance threshold.

## Results

### Ocular mucosal immunization increased anti-PmpC IgA levels in sera

**Fig 1:**
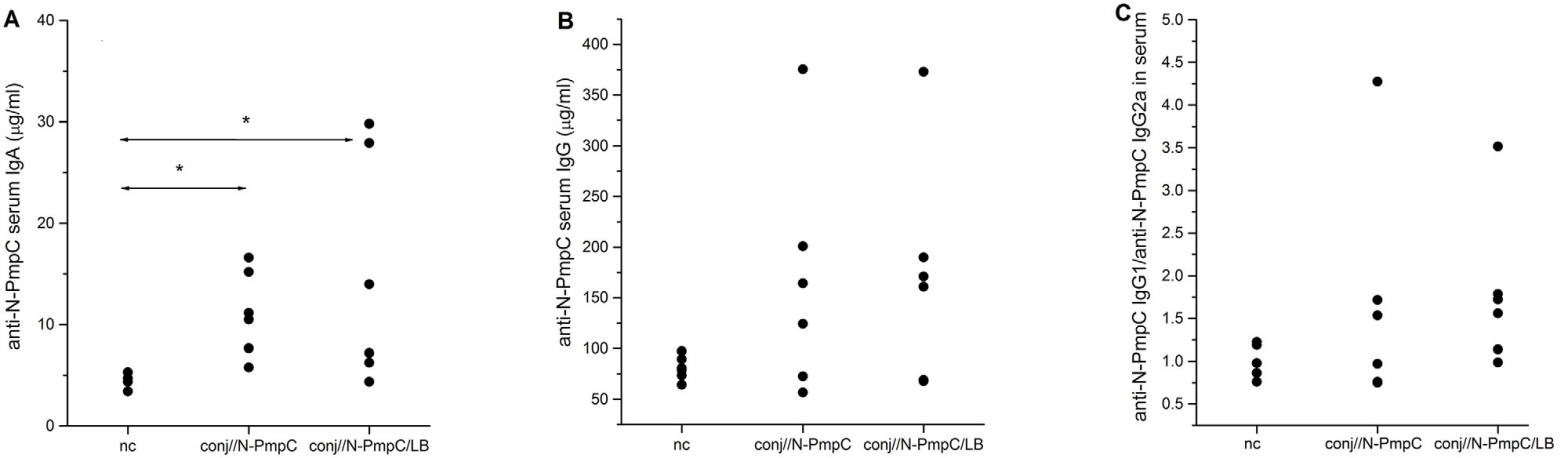
Anti-PmpC antibody serum levels. Levels of (A) anti-N-PmpC IgA and (B) anti-N-PmpC IgG and (C) ratio of the levels of N-PmpC-specific IgG1 and IgG2a antibodies in the serum of BALB/c mice immunized via the conjunctiva and age-matched normal controls (nc). All serum samples were collected two weeks after completion of the indicated immunization protocol and were assayed by ELISA. Results for each individual serum sample are presented. The levels of N-PmpC-specific IgG1 and IgG2a were judged according to the A_492/620_ values recorded for individual serum samples. The statistical significance of the observed differences was evaluated using Kruskal -Wallis test followed by Dunn's multiple comparisons test to compare between groups (*P* < 0.05*, *P*<0.005**). Compared groups are indicated by two-head arrow.

Ocular immunization with N-PmpC, either alone or formulated with LB, promoted a systemic rise in the level of N-PmpC-specific IgA vs the nc group (Fig 1A; *P<*0.005 for conj//N-PmpC, *P<*0.05 for conj//N-PmpC/LB). However, no significant difference was found between conj// groups in the levels of all antibodies analysed. The mean serum anti-N-PmpC IgA concentrations recorded in the nc, conj//N-PmpC and conj//N-PmpC/LB groups were 4.25±0.30 μg/ml, 11.14±1.71 μg/ml and 14.91±4.62 μg/ml, respectively.

Analysis of anti-N-PmpC IgG showed no significant difference between the conj// groups (Fig 1B). However, the mean levels of anti-N-PmpC IgG were higher in the serum of the conj//N-PmpC and conj//N-PmpC/LB groups (165.68±47.42 μg/ml and 171.93±45.56 μg/ml, respectively) vs the nc group (80.67±4.76 μg/ml). In addition, subclass level analyses showed no significant difference in the contribution of N-PmpC-specific IgG1 and IgG2a antibodies in both conj// groups vs the nc group (Fig 1C).

### N-PmpC-specific SIgA levels in mucosal washes were elevated significantly following immunization

**Fig 2:**
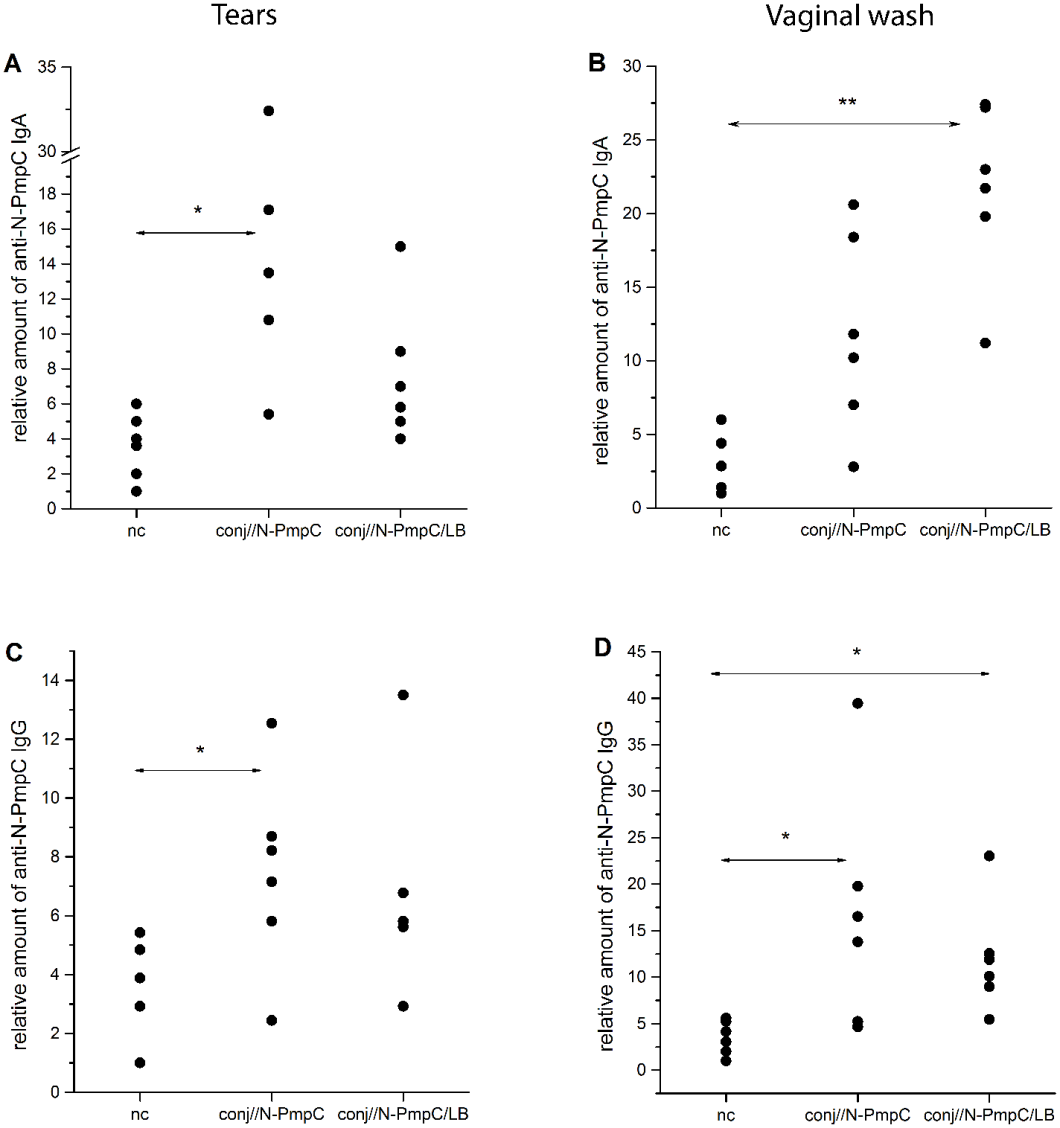
Anti-PmpC antibody levels from mucosal surfaces. (A-B) Anti-N-PmpC IgA levels and (C-D) anti-N-PmpC IgG levels in the tears (A and C) and vaginal washes (B and D) of BALB/c mice immunized via the conjunctiva and age-matched controls (nc). All samples were collected two weeks after the completion of the indicated immunization protocol and were assayed by ELISA. Results for each individual sample are presented. The statistical significance of the observed differences was evaluated using Kruskal-Wallis test followed by Dunn's multiple comparisons test to compare between groups (*P* < 0.05*, *P*<0.005**). Compared groups are indicated by two-head arrow.

An evaluation of N-PmpC-specific antibodies in the tears and vaginal washes generally showed a rise in the local concentrations of both N-PmpC-specific IgG and IgA after N-PmpC immunization via the ocular mucosa (Fig 2). The concentration of N-PmpC-specific SIgA was significantly higher in the wash samples from both conj// groups vs the nc group (Fig 2A and 2B). In addition, an elevated level of specific SIgA was found in the vaginal wash samples from the conj//N-PmpC/LB group compared with the conj//N-PmpC group (Fig 2B; *P*<0.05).

The N-PmpC-specific IgG concentration was also elevated in the wash samples collected from conj// mice compared with nc mice (Fig 2C and 2D). A significant rise in anti-N-PmpC IgG level was observed in the tears of conj//N-PmpC mice (Fig 2C; *P*<0.05 vs nc) and in the vaginal washes collected from both conj// groups vs the nc group (Fig 2D; *P*<0.05 for both groups). Comparison of anti-N-PmpC IgG levels in the washes collected at the same mucosal surface from the conj//N-PmpC and conj//N-PmpC/LB mice revealed no significant differences.

### Anti-CtB antibody levels in sera, tears and vaginal washes were increased significantly following immunization

**Fig 3:**
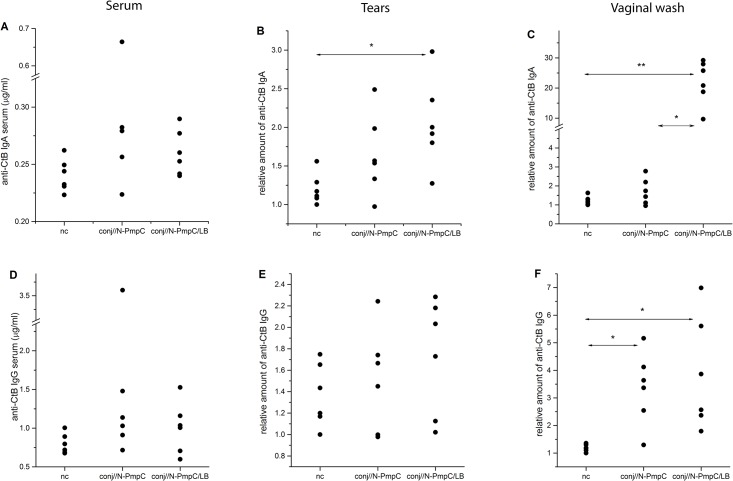
Anti-CtB antibody levels in serum and on mucosal surfaces. (A-C) Anti-CtB IgA levels and (C-E) anti-CtB IgG levels in the serum (A and D), tears (B and E) and vaginal washes (C and F) of BALB/c mice immunized via the conjunctiva and age-matched controls (nc). All samples were collected two weeks after the completion of the indicated immunization protocol and were assayed by ELISA. Results for each individual sample are presented. The statistical significance of the observed differences was evaluated using Kruskal-Wallis test followed by Dunn's multiple comparisons test to compare between groups (*P* < 0.05*, *P*<0.005**). Compared groups are indicated by two-head arrow.

IgG and IgA analysis of sera and washes collected from mucosal surfaces revealed that conj// immunization with N-PmpC promoted the production of both CtB-specific immunoglobulins (Fig 3).

The mean concentration of anti-CtB antibodies in the sera of the nc, conj//N-PmpC and conj//N-PmpC/LB mice were 0.24±0.01 μg/ml, 0.34±0.07 μg/ml and 0.26±0.01 μg/ml, respectively, for IgA (Fig 3A), and 0.80±0.05 μg/ml, 1.48±0.44 μg/ml and 1.01±0.14 μg/ml, respectively, for IgG (Fig 3D).

The most prominent increase in anti-CtB antibody levels after either formulation of conjunctival N-PmpC immunization was recorded in the vaginal washes. Indeed, the conj//N-PmpC group produced more vaginal anti-CtB IgG (Fig 3F; *P* < 0.005 vs nc), whereas the conj//N-PmpC/LB group produced more of both vaginal anti-CtB IgA (Fig 3C; *P* < 0.0009 vs nc) and anti-CtB IgG (Fig 3D; *P* < 0.05 vs nc). Compared with the group immunized with N-PmpC alone, LB co-administration exerted a positive effect on vaginal CtB-specific SIgA levels (Fig 3C; *P*<0.005).

LB co-administration exerted a significant positive effect on CtB-specific SIgA level in tears as well (Fig 3B; *P*<0.05 vs nc).

### N-PmpC immunization promoted effector T cell differentiation in SMLNs

**Fig 4:**
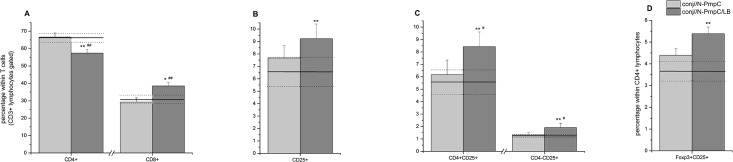
Phenotyping of SMLN T lymphocytes by FACS analysis. Bar graph showing the contribution (expressed in %) of specific T cell populations to the overall T cell pool within SMLNs isolated from BALB/c mice two weeks after the completion of the indicated immunization protocol. The mean percentages of (A) CD4+ and CD8+, (B) CD25+, and (C) CD4-CD25+ and CD4+CD25+ cells within T lymphocytes pool are presented. Lymphocytes were gated according to their position within the forward scatter (FSC) vs side scatter (SSC) and T cells were marked according to the expression of CD3+ (S1 Fig). Assessment of Foxp3 and CD25 co-expression was performed for CD4+ lymphocytes (S2 Fig) and mean percentages of Foxp3+CD25+ within CD4+ lymphocytes (D) are presented. The mean percentage of corresponding T cell population recorded in SMLN isolated from age-matched control mice (nc) is presented on each graph as a solid line (mean value) and dotted lines (upper and lower standard error values). The statistical significance of the observed differences was evaluated using Kruskal-Wallis test followed by Dunn's multiple comparisons test to compare between groups (immunized vs nc *P* < 0.05*, *P*<0.005**; between immunized groups *P* < 0.05^#^, *P*<0.005^##^).

Analysis of the SMLN T cell pool (i.e., CD3+ cells within the lymphocyte gate, S1 Fig) revealed that conjunctival immunization with N-PmpC in the presence of LB promoted the expansion of CD8+ T cells over the CD4+ (Fig 4A, percentage of CD8+ T cells in conj//N-PmpC/LB, *P*<0.05 vs nc). In addition, the percentage of CD25+ T cells in conj//N-PmpC LB was markedly increased compared with the nc group (Fig 4B, *P<*0.005). The increased percentage of CD25+ T lymphocytes was mainly due to the higher abundance of CD3+CD4+CD25+ lymphocytes (Fig 4C, conj//N-PmpC/LB, *P*<0.005 vs nc).

Simultaneous analysis of CD4, CD25 and Foxp3 expression on SMLN lymphocytes (S2 Fig) revealed a significantly lower percentage of CD4+CD25+Foxp3+ cells (Treg) in the nc group than in the conj//N-PmpC/LB mice (Fig 4D, *P<*0.005).

### Chlamydia antigens stimulated in vitro proliferation of SMLN cells

**Fig 5:**
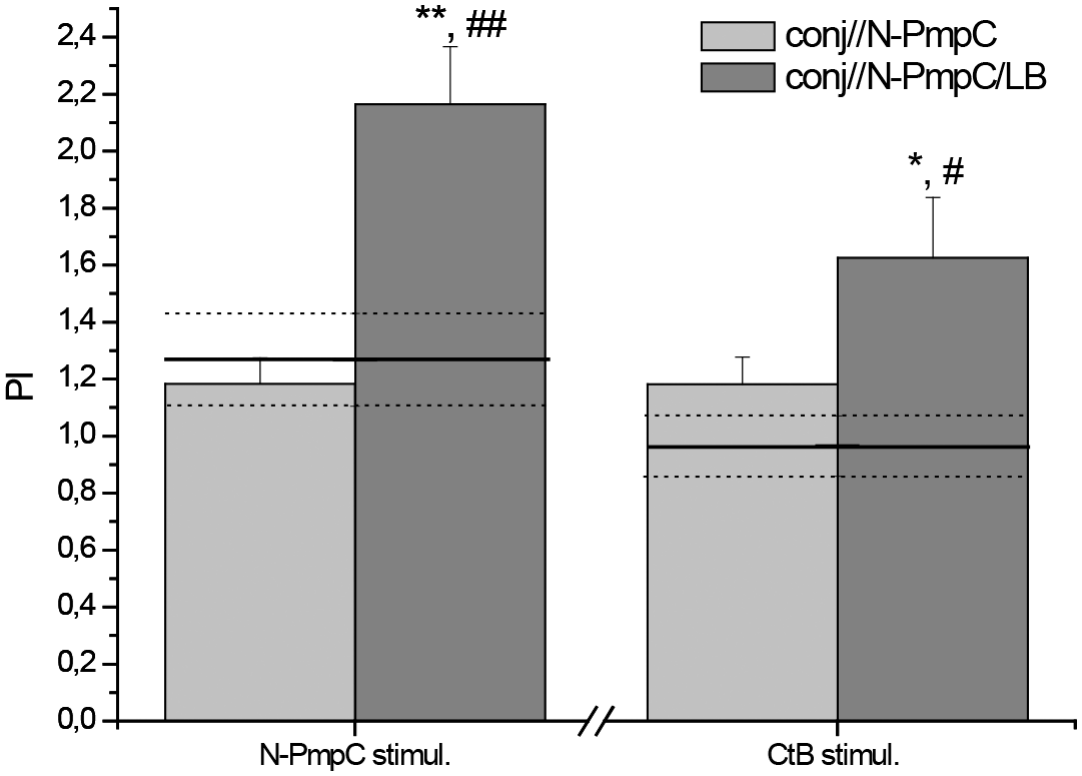
*In vitro* analysis of proliferation of primary SMLN cells subjected to N-PmpC and CtB stimulation. Bar graph showing the proliferation indices (PI) of N-PmpC- and CtB-stimulated SMLN cells isolated from BALB/c mice immunized via the conjunctiva. The proliferation indices of SMLN cells isolated from age-matched control mice (nc) are presented on the graph as a solid line (mean value) and dotted lines (upper and lower standard error values). The number of viable SMLN cells was assessed using the Cell Counting Kit*-*8 following a 48 h culture period in 10% FCS/50 μM β-mercaptoethanol/RPMI 1640 medium supplemented or not with the indicated stimulator (10 μg/ml for N-PmpC or 1x10^6^ CFU/ml for CtB). PIs were calculated for each mouse. The results are presented as the mean PIs ± SE for each experimental group (n = 6). The statistical significance of the observed differences was evaluated using Kruskal-Wallis test followed by Dunn's multiple comparisons test to compare between groups (immunized vs nc *P* < 0.05*, *P*<0.005**; between immunized groups *P* < 0.05^#^, *P*<0.005^##^).

With the aim of evaluating local immune responses in mice immunized with N-PmpC via the conjunctiva, SMLN cells were stimulated *in vitro* with the N-PmpC and CtB antigens (Fig 5). Irrespective of the stimulator (N-PmpC or CtB), the proliferation of SMLN cells isolated from the conj//N-PmpC/LB group was significantly higher than that of SMLN cells from both corresponding nc and conj//N-PmpC groups (N-PmpC stimulation: *P*<0.005; CtB stimulation: *P*<0.05).

### LB adjuvant affected the cytokine pattern of SMLN cells and their response to Ct antigen stimulation

**Fig 6:**
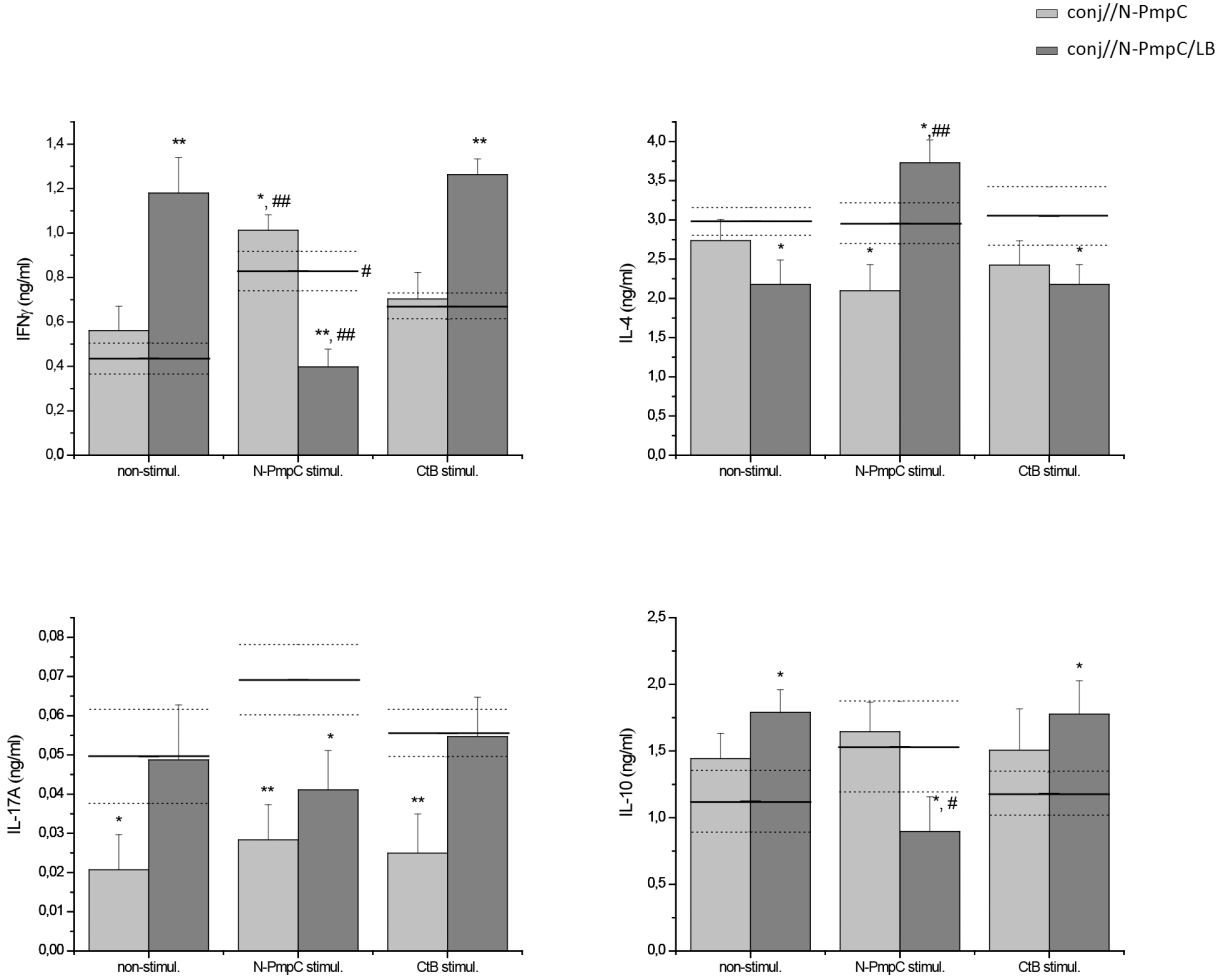
Effect of N-PmpC and CtB stimulation on the *in vitro* cytokine pattern in SMLN cells isolated after ocular mucosal immunization with N-PmpC and N-PmpC/LB. Bar graphs representing the levels of IFNγ, IL-4, IL-10 and IL-17A in the supernatants of non-stimulated, N-PmpC- or CtB-stimulated SMLN cells isolated from BALB/c mice immunized via the conjunctiva (bars). The corresponding measurements in SMLN cells isolated from age-matched controls (nc) are presented on the graphs as a solid line (mean value) and dotted lines (upper and lower standard error values). SMLN cells were cultured at 37°C under a 5% CO_2_ atmosphere for 48 h in 10% FCS/50 μM β-mercaptoethanol/RPMI 1640 medium supplemented or not with the indicated stimulator (10 μg/ml for N-PmpC or 1x10^6^ CFU/ml for CtB). The results are presented as the mean concentrations ± SE (n = 6). The statistical significance of the observed differences was evaluated using Kruskal-Wallis test followed by Dunn's multiple comparisons test to compare between groups (immunized vs nc stimulated in a same way *P* < 0.05*, *P*<0.005**; vs non-stimulated samples of the same group *P* < 0.05^#^, *P*<0.005^##^).

The basal *in vitro* production of effector cytokines (IFN-γ as Th1 marker, IL-4 as Th2 marker, IL-17A as Th17 marker) and the regulatory cytokine IL-10, recorded in non-stimulated SMLN cultures, differed among conjunctively immunized groups and was altered compared with the nc group (Fig 6).

Compared with the nc non-stimulated SMLN cells, the conj//N-PmpC non-stimulated SMLN cells produced slightly more IFN-γ and IL-10, whereas the secretion of IL-17A was significantly reduced (*P*<0.05). The basal IFN-γ and IL-10 production levels were highest in the SMLN cultures from the conj//N-PmpC/LB group (IFN-γ: *P*<0.005 vs nc; IL-10: *P*<0.005 vs nc) and were accompanied by a significant reduction in IL-4 secretion (*P*<0.05 vs nc). SMLN cells from the conj//N-PmpC/LB group without any stimulation secreted IL-17A in amounts comparable with those recorded in non-stimulated nc SMLN cells.

In nc SMLN cultures, N-PmpC stimulation enhanced the secretion of IFN-γ (*P*<0.05 vs non-stimulated nc), IL-10 and IL-17A but did not affect the secretion of IL-4. Compared with the corresponding non-stimulated cultures, N-PmpC stimulation enhanced the production of IFN-γ (*P*<0.005), IL-17A and IL-10, and inhibited IL-4 secretion in conj//N-PmpC SMLN cells. In contrast, N-PmpC stimulation decreased IL-17A, IFN-γ (*P<*0.005 vs non-stimulated) and IL-10 (*P<*0.05 vs non-stimulated) production while enhanced IL-4 secretion (*P<*0.05 vs non-stimulated) in conj//N-PmpC/LB SMLN cells.

CtB stimulation had less impact on the overall production of all tested cytokines. Compared with the corresponding non-stimulated cultures, CtB stimulation did not significantly affect the production of the tested cytokines. However, in the CtB-stimulated conj//N-PmpC/LB cultures compared with the CtB-stimulated nc SMLN cultures, the productions of IFNγ (*P<*0.005) and IL-10 (*P<*0.05) were significantly higher while IL-4 production was decreased (*P<*0.05).

## Discussion

The administration of the N-PmpC chlamydia-specific subunit antigen via the ocular mucosa elicited antigen specific humoral and cell-mediated immune responses.

The importance of the humoral immune response in the protection against Ct has been debated extensively [24–26]. Compared with immunization with N-PmpC alone, LB co-administration had no significant impact on specific serum antibodies. Interestingly, the levels of chlamydial specific SIgAs were significantly higher in the vaginal washes of mice immunized with N-PmpC/LB vs mice immunized with N-PmpC alone. This finding shows that ocular mucosal immunization could increase antigen-specific antibodies in distant mucosal compartments, such as the vaginal mucosa, when antigen is presented to the immune system within an adequate adjuvant delivery system. This might be important because subunit formulations with different adjuvants delivered into the vagina were shown to be poorly immunogenic due to immunological properties of the female reproductive tract during the menstrual cycle [27, 28]. The mechanism behind this warrants further attention and will be explored in our further experiments.

The fact that anti-N-PmpC antibody levels changed in parallel with corresponding (same isotype and location) anti-CtB antibodies levels implies that the observed anti-CtB reactivity is likely due to cross-reactivity between anti-N-PmpC and anti-CtB antibodies. This suggests that the antibodies secreted upon N-recombinant PmpC stimulation might 1) recognize naturally occurring antigens and 2) cross-react with the PmpC fragment of Ct serovars that were not the source the recombinant antigen.

It is known that the cellular immune response is important for protection against Ct infection [29–31]. IFN-γ, known as the key molecule in the protection against Ct invasion, can be produced by both Th1 (CD4+) and Tc1 (CD8+) cells [24]. However, CD4+ T cell depletion was shown to abrogate the protection against Ct-infection more profoundly than the depletion of CD8+ T cells [32]. In our study, LB promoted the expansion of CD8+ T cells and Th effector (CD3+CD4+CD25+) cells compared with the conj//N-PmpC group. The elevated percentage of CD8+ cells within the SMLN T cells of conj//N-PmpC/LB mice is in line with the results of MHC I/proteasome cleavage prediction testing, which suggested that N-PmpC-derived peptides could be presented in the context of MHC I [32]. The abovementioned findings, together with the strong proliferative response observed in conj//N-PmpC/LB SMLN cells upon CtB stimulation, suggest that LB or similar probiotic adjuvants may be promising tools for the development of efficient anti-Ct vaccines. Moreover, cytokine production analysis in *ex vivo* SMLN cultures suggests that N-PmpC administration via the conjunctiva promoted a slight Th1 immune response, which was prominently enhanced by LB co-administration. Further, the comparison of SMLN cell responses to *in vitro* N-PmpC and CtB stimulations confirms that the applied immunization protocol influences not only the intensity but also the quality of the response to subsequent antigen stimulation. Results we got *in vitro* imply that in N-PmpC/LB-immunized mice subsequent stimulation with N-PmpC alone would not be able to enhance Th1 immune response. Even more, it is likely that Th1 specific immune response would be inhibited. This result suggests that for an eventual booster immunization N-PmpC also has to be formulated with some adjuvant capable to stimulate innate immunity, in order to tackle natural Ct infection. However, during naturally occurring infection PmpC is not alone i.e. it is in the context of chlamydia elementary bodies. Accordingly, we consider the results gotten upon CtB stimulation more relevant for the prediction of susceptibility to infection.

We also demonstrated that the increased percentage of effector T cells was accompanied by an expansion of the Treg cell population, and that, in all cases, enhanced IFN-γ secretion was paralleled by enhanced IL-10 production. However, the activation of regulatory mechanisms following vaccination may be a double-edged sword [25]. This response may represent a beneficial self-limiting mechanism preventing a strong inflammatory immune response, thereby diminishing pathological sequelae and prevent potentially harmful autoreactive immune responses through molecular mimicry [33]. However, an increase in the number of Foxp3+ Tregs has been shown to prevent the development of an efficient immune response upon immunization [24]. Besides, one of the inherent characteristics of Foxp3+ T cells is “plasticity” i.e. they can undergone reprogramming in the sense of acquisition of an effector phenotype [34]. It was shown by others that stimulation of rabbit Tregs through TLR2 decreased their immunosuppressive potential and promoted the concomitant expansion of T effector cells [21]. Similar findings were reported in mice [35, 36]. Although Foxp3 expression is considered a marker of the Treg population and implies on cells capable of exerting suppressive impact, the real suppressive potential of Tregs in this particular situation (i.e. their functional status) cannot be discerned by Foxp3 expression only. For all these reasons, the actual immunosuppressive potential of Tregs during our immunization protocol should be evaluated in more detail.

Although the precise mechanisms by which probiotics act as adjuvants remain unknown, an immunoenhancing strain of *Lactobacillus* (*L*. *rhamnosus* HN001) identified by Gill et al and isolated originally from Cheddar cheese, compared with placebo, was shown to significantly induce phagocytic cell activity and anti-cholera specific antibody production in gut mucosa when delivered simultaneously with a cholera toxin vaccine [37]. The proposed mechanisms of action for the adjuvant activity of probiotics include an influence on innate immune cells, such as intestinal macrophages or dendritic cells, which may in turn enhance antigen presentation and promote the preferential differentiation of mucosal lymphocytes towards the production of protective antibodies particularly at sites where a multitude of antigens are encountered constantly, such as in the conjunctiva- and vagina-associated lymphoid tissues [38].

In conclusion, we provided evidence that ocular mucosal immunization with combined use of a specific antigen and a probiotic stimulates specific immune responses not only locally but also in the vaginal mucosa.

## Supporting Information

S1 Fig Phenotyping of SMLN lymphocytes by FACS analysis. Lymphocytes were gated according to their position within the forward scatter (FSC) vs side scatter (SSC) plots and analysed for the percentage of T (CD3+CD19-) and B (CD3-CD19+) cells. T cells (gated CD3+ lymphocytes) were further analysed for the expression of CD4, CD8 and CD25. Cells were analysed using a BD FACScan^™^flow cytometer (BD Biosciences) and BD CellQuest^™^ software. Representative dot plots and histograms are presented.
(TIF)

S2 Fig Phenotyping of SMLN Treg lymphocytes by FACS analysis. The co-expression of CD4, CD25 and Foxp3 on lymphocytes from SMLN of BALB/c mice immunized via the conjunctiva and age-matched controls (nc) was analysed. Lymphocytes were gated according to their position within the FSC vs SSC plots. Then, CD4+ lymphocytes were gated and further analysed for the expression of CD25 and Foxp3. Cells were analysed using a BD FACScan^™^flow cytometer (BD Biosciences) and BD CellQuest^™^ software. Representative dot plots and histograms are presented.
(TIF)

## References

[1] Seo KY, Han SJ, Cha HR, Seo SU, Song JH, Chung SH, et al Eye mucosa: an efficient vaccine delivery route for inducing protective immunity. J Immunol. 2010;185:3610–9. 10.4049/jimmunol.1000680 20709955

[2] Nesburn AB, Ramos TV, Zhu X, Asgarzadeh H, Nguyen V, BenMohamed L. Local and systemic B cell and Th1 responses induced following ocular mucosal delivery of multiple epitopes of herpes simplex virus type 1 glycoprotein D together with cytosine-phosphate-guanine adjuvant. Vaccine. 2005;23:873–83. 15603887 10.1016/j.vaccine.2004.08.019

[3] Mitragotri S. Immunization without needles. Nat Rev Immunol. 2005;5:905–16. 16239901 10.1038/nri1728

[4] Schachter J, Caldwell HD. Chlamydiae. Annual review of microbiology. 1980;34:285–309. 7002026 10.1146/annurev.mi.34.100180.001441

[5] Taylor HR, Burton MJ, Haddad D, West S, Wright H. Trachoma. Lancet. 2014;384:2142–52. 10.1016/S0140-6736(13)62182-0 25043452

[6] Norman J. Epidemiology of female genital Chlamydia trachomatis infections. Best Pract Res Clin Obstet Gynaecol. 2002;16:775–87. 12473281 10.1053/beog.2002.0325

[7] Schachter J. Overview of Chlamydia trachomatis infection and the requirements for a vaccine. Rev Infect Dis. 1985;7:713–6. 3840910 10.1093/clinids/7.6.713

[8] Hafner LM, Wilson DP, Timms P. Development status and future prospects for a vaccine against Chlamydia trachomatis infection. Vaccine. 2014;32:1563–71. 10.1016/j.vaccine.2013.08.020 23973245

[9] Cochrane M, Armitage CW, O'Meara CP, Beagley KW. Towards a Chlamydia trachomatis vaccine: how close are we? Future microbiology. 2010;5:1833–56. 10.2217/fmb.10.148 21155665

[10] Kari L, Whitmire WM, Olivares-Zavaleta N, Goheen MM, Taylor LD, Carlson JH, et al A live-attenuated chlamydial vaccine protects against trachoma in nonhuman primates. J Exp Med. 2011;208:2217–23. 10.1084/jem.20111266 21987657 PMC3201208

[11] Brunham RC, Rappuoli R. Chlamydia trachomatis control requires a vaccine. Vaccine. 2013;31:1892–7. 10.1016/j.vaccine.2013.01.024 23375977 PMC4148049

[12] Gray RT, Beagley KW, Timms P, Wilson DP. Modeling the impact of potential vaccines on epidemics of sexually transmitted Chlamydia trachomatis infection. J Infect Dis. 2009;199:1680–8. 10.1086/598983 19432550

[13] Wehrl W, Brinkmann V, Jungblut PR, Meyer TF, Szczepek AJ. From the inside out—processing of the Chlamydial autotransporter PmpD and its role in bacterial adhesion and activation of human host cells. Molecular Microbiology. 2004;51:319–34. 14756775 10.1046/j.1365-2958.2003.03838.x

[14] Crane DD, Carlson JH, Fischer ER, Bavoil P, Hsia RC, Tan C, et al Chlamydia trachomatis polymorphic membrane protein D is a species-common pan-neutralizing antigen. Proc Natl Acad Sci U S A. 2006;103:1894–9. 16446444 10.1073/pnas.0508983103 PMC1413641

[15] Molleken K, Schmidt E, Hegemann JH. Members of the Pmp protein family of Chlamydia pneumoniae mediate adhesion to human cells via short repetitive peptide motifs. Molecular microbiology. 2010;78:1004–17. 10.1111/j.1365-2958.2010.07386.x 21062373 PMC2997323

[16] Rockey DD, Lenart J, Stephens RS. Genome sequencing and our understanding of chlamydiae. Infection and immunity. 2000;68:5473–9. 10992442 10.1128/iai.68.10.5473-5479.2000 PMC101494

[17] Schautteet K, De Clercq E, Vanrompay D. Chlamydia trachomatis vaccine research through the years. Infect Dis Obstet Gynecol. 2011;2011:963513 10.1155/2011/963513 21747646 PMC3124257

[18] Boje S, Olsen AW, Erneholm K, Agerholm JS, Jungersen G, Andersen P, et al A multi-subunit Chlamydia vaccine inducing neutralizing antibodies and strong IFN-gamma CMI responses protects against a genital infection in minipigs. Immunol Cell Biol. 2015. 10.1038/icb.2015.79 PMC4748142 26268662

[19] Inic-Kanada A, Stojanovic M, Schlacher S, Stein E, Belij-Rammerstorfer S, Marinkovic E, et al Delivery of a Chlamydial Adhesin N-PmpC Subunit Vaccine to the Ocular Mucosa Using Particulate Carriers. PLoS One. 2015;10:e0144380 10.1371/journal.pone.0144380 26656797 PMC4684359

[20] Talreja J, Dileepan K, Puri S, Kabir MH, Segal DM, Stechschulte DJ, et al Human conjunctival epithelial cells lack lipopolysaccharide responsiveness due to deficient expression of MD2 but respond after interferon-gamma priming or soluble MD2 supplementation. Inflammation. 2005;29:170–81. 17093906 10.1007/s10753-006-9014-y

[21] Dasgupta G, Chentoufi AA, You S, Falatoonzadeh P, Urbano LA, Akhtarmalik A, et al Engagement of TLR2 reverses the suppressor function of conjunctiva CD4+CD25+ regulatory T cells and promotes herpes simplex virus epitope-specific CD4+CD25- effector T cell responses. Invest Ophthalmol Vis Sci. 2011;52:3321–33. 10.1167/iovs.10-6522 21273544 PMC3109031

[22] Iovieno A, Lambiase A, Sacchetti M, Stampachiacchiere B, Micera A, Bonini S. Preliminary evidence of the efficacy of probiotic eye-drop treatment in patients with vernal keratoconjunctivitis. Graefes Arch Clin Exp Ophthalmol. 2008;246:435–41. 18040708 10.1007/s00417-007-0682-6

[23] Becker E, Hegemann JH. All subtypes of the Pmp adhesin family are implicated in chlamydial virulence and show species-specific function. MicrobiologyOpen. 2014;3:544–56. 10.1002/mbo3.186 24985494 PMC4287181

[24] Brunham RC, Rey-Ladino J. Immunology of Chlamydia infection: implications for a Chlamydia trachomatis vaccine. Nat Rev Immunol. 2005;5:149–61. 15688042 10.1038/nri1551

[25] Bailey RL, Kajbaf M, Whittle HC, Ward ME, Mabey DC. The influence of local antichlamydial antibody on the acquisition and persistence of human ocular chlamydial infection: IgG antibodies are not protective. Epidemiology and infection. 1993;111:315–24. 8405158 10.1017/s0950268800057022 PMC2271382

[26] Li Z, Palaniyandi S, Zeng R, Tuo W, Roopenian DC, Zhu X. Transfer of IgG in the female genital tract by MHC class I-related neonatal Fc receptor (FcRn) confers protective immunity to vaginal infection. Proc Natl Acad Sci U S A. 2011;108:4388–93. 10.1073/pnas.1012861108 21368166 PMC3060240

[27] Russell MW. Immunization for protection of the reproductive tract: a review. Am J Reprod Immunol. 2002;47:265–8. 12148540 10.1034/j.1600-0897.2002.01099.x

[28] Rosenthal KL, Gallichan WS. Challenges for vaccination against sexually-transmitted diseases: induction and long-term maintenance of mucosal immune responses in the female genital tract. Semin Immunol. 1997;9:303–14. 9327525 10.1006/smim.1997.0086

[29] Perry LL, Su H, Feilzer K, Messer R, Hughes S, Whitmire W, et al Differential sensitivity of distinct Chlamydia trachomatis isolates to IFN-gamma-mediated inhibition. J Immunol. 1999;162:3541–8. 10092812

[30] Gondek DC, Olive AJ, Stary G, Starnbach MN. CD4+ T cells are necessary and sufficient to confer protection against Chlamydia trachomatis infection in the murine upper genital tract. J Immunol. 2012;189:2441–9. 10.4049/jimmunol.1103032 22855710 PMC3690950

[31] Johansson M, Schon K, Ward M, Lycke N. Genital tract infection with Chlamydia trachomatis fails to induce protective immunity in gamma interferon receptor-deficient mice despite a strong local immunoglobulin A response. Infect Immun. 1997;65:1032–44. 9038313 10.1128/iai.65.3.1032-1044.1997 PMC175085

[32] Wizel B, Nystrom-Asklin J, Cortes C, Tvinnereim A. Role of CD8(+)T cells in the host response to Chlamydia. Microbes Infect. 2008;10:1420–30. 10.1016/j.micinf.2008.08.006 18790073 PMC2640455

[33] Hu VH, Holland MJ, Burton MJ. Trachoma: Protective and Pathogenic Ocular Immune Responses to *Chlamydia trachomatis*. PLoS Negl Trop Dis. 2013;7:e2020 10.1371/journal.pntd.0002020 23457650 PMC3573101

[34] Mellor AL, Munn DH. Physiologic control of the functional status of Foxp3+ regulatory T cells. J Immunol. 2011;186:4535–40. 10.4049/jimmunol.1002937 21464094 PMC3808246

[35] Liu H, Komai-Koma M, Xu D, Liew FY. Toll-like receptor 2 signaling modulates the functions of CD4+ CD25+ regulatory T cells. Proc Natl Acad Sci U S A. 2006;103:7048–53. 16632602 10.1073/pnas.0601554103 PMC1444884

[36] Sutmuller RP, den Brok MH, Kramer M, Bennink EJ, Toonen LW, Kullberg BJ, et al Toll-like receptor 2 controls expansion and function of regulatory T cells. The Journal of clinical investigation. 2006;116:485–94. 16424940 10.1172/JCI25439 PMC1332026

[37] Gill HS, Rutherfurd KJ. Viability and dose-response studies on the effects of the immunoenhancing lactic acid bacterium Lactobacillus rhamnosus in mice. The British journal of nutrition. 2001;86:285–9. 11502243 10.1079/bjn2001402

[38] Licciardi PV, Tang ML. Vaccine adjuvant properties of probiotic bacteria. Discovery medicine. 2011;12:525–33. 22204769

