# Supplementary figures and images for: A Probiotic Adjuvant Lactobacillus rhamnosus Enhances Specific Immune Responses after Ocular Mucosal Immunization with Chlamydial Polymorphic Membrane Protein C
Source: PLoS One. 2016 Sep 16;11(9):e0157875. doi: 10.1371/journal.pone.0157875 (PMC5026373; doi:10.1371/journal.pone.0157875)

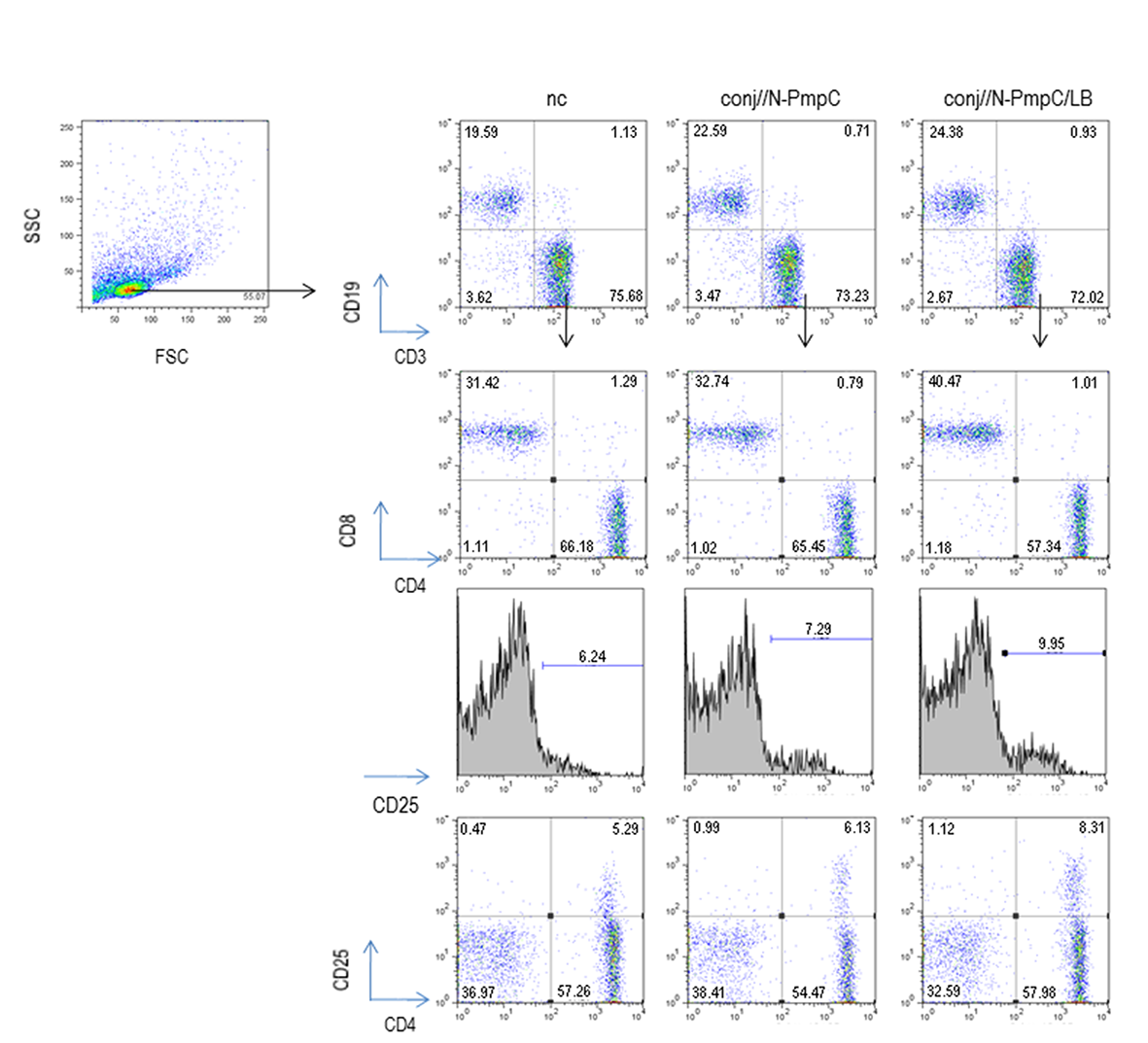

Supplement: S1 Fig — Lymphocytes were gated according to their position within the forward scatter (FSC) vs side scatter (SSC) plots and analysed for the percentage of T (CD3+CD19-) and B (CD3-CD19+) cells. T cells (gated CD3+ lymphocytes) were further analysed for the expression of CD4, CD8 and CD25. Cells were analysed using a BD FACScan™flow cytometer (BD Biosciences) and BD CellQuest™ software. Representative dot plots and histograms are presented. (TIF) [file pone.0157875.s001.tif]

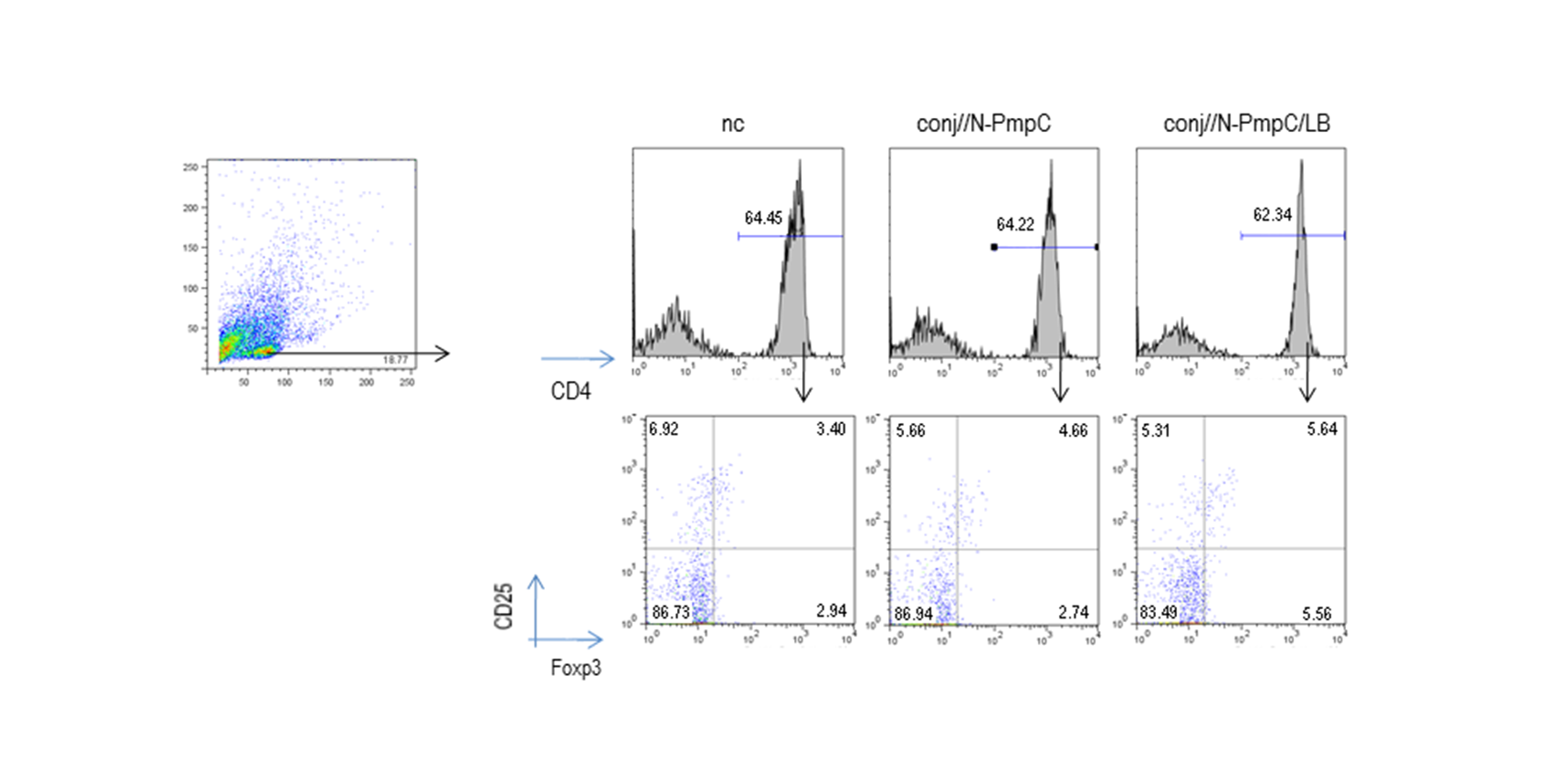

Supplement: S2 Fig — The co-expression of CD4, CD25 and Foxp3 on lymphocytes from SMLN of BALB/c mice immunized via the conjunctiva and age-matched controls (nc) was analysed. Lymphocytes were gated according to their position within the FSC vs SSC plots. Then, CD4+ lymphocytes were gated and further analysed for the expression of CD25 and Foxp3. Cells were analysed using a BD FACScan™flow cytometer (BD Biosciences) and BD CellQuest™ software. Representative dot plots and histograms are presented. (TIF) [file pone.0157875.s002.tif]
